# Transcriptomics of circulating neutrophils in dairy cows with subclinical hypocalcemia

**DOI:** 10.3389/fvets.2022.959831

**Published:** 2022-09-13

**Authors:** Bingbing Zhang, Xinru Ma, Baoyin Huang, Qianming Jiang, Juan J. Loor, Xinquan Lv, Wei Zhang, Ming Li, Jianan Wen, Yufeng Yin, Jingjing Wang, Wei Yang, Chuang Xu

**Affiliations:** ^1^College of Life Science and Technology, Heilongjiang Bayi Agricultural University, Daqing, China; ^2^College of Animal Science and Veterinary Medicine, Heilongjiang Bayi Agricultural University, Daqing, China; ^3^Animal Husbandry and Veterinary Branch of Heilongjiang Academy of Agricultural Sciences, Qiqihaer, China; ^4^Mammalian NutriPhysioGenomics, Division of Nutritional Sciences, Department of Animal Sciences, University of Illinois, Urbana, IL, United States; ^5^College of Veterinary Medicine, China Agricultural University, Beijing, China

**Keywords:** PMNL, RNA sequencing, transition, dairy cows, hypocalcemia

## Abstract

Hypocalcemia is closely associated with inflammatory diseases in dairy cows. Recent research has underscored the key role of calcium in the adaptations of the innate immune system during this period. The main objective in the present study was to compare the transcriptome profiles and analyze differences in the expression of neutrophil (PMNL) immune function-related genes and calcium binding-related genes in hypocalcemic cows. At 2 days postpartum, a concentration >2.10 mmol Ca^2+^/L was used to classify cows as controls (CON), and a concentration <2.00 mmol Ca^2+^/L used to classify cows as low-calcium (LCAL) (*n* = 8 in each group). A routine medical examination was conducted by the attending veterinarian to ensure there were no other complications and that the blood β-hydroxybutyrate was <1.2 mmol/L. Blood was collected from the tail vein (20 mL) to isolate PMNL, and 5 cows in each group were used for RNA sequencing and statistical analysis of gene expression differences. Transcriptome RNA-seq sequencing analysis was *via* omicsstudio using the R package edgeR. GO and KEGG enrichment analysis were used for bioinformatics. The remaining 3 cows in each group were used for validation of RNA sequencing data *via* quantitative PCR, which confirmed the observed responses. Compared with CON, 158 genes in LCAL were significantly up-regulated and 296 genes were down-regulated. The downregulation of Interleukin-12 (CXCL12), Tubulin beta chain (TUBB1), L1 cell adhesion molecule (L1CAM), and Myeloperoxidase (MPO) indicated a decrease in immune function of PMNL in LCAL cows. The decreased expression of calcium-binding pathway-related genes in PMNL of LCAL cows indicated a decrease in immune function of PMNL likely related to calcium ions. For example, cartilage acid protein 1 (CRTAC1) and calcium/calmodulin-dependent kinase 4 (CAMK4) were significantly reduced in LCAL cows. The upregulation of Cyclin dependent kinase inhibitor 1A (CDKN1A), Perforin 1 (PRF1), and Homeodomain interacting protein kinase 3 (HIPK3) indicated that LCAL led to greater cell apoptosis and senescence. Overall, the analyses indicated that the reduction in PMNL immune function during hypocalcemia is associated with downregulation of intracellular Ca^2+^ related genes and upregulation of genes controlling apoptosis and senescence. Together, these alterations contribute to an immunosuppressive state during the transition period.

## Introduction

Subclinical hypocalcemia is a relatively common disorder in high-producing multiparous dairy cows during the transition from late-pregnancy to lactation. It can lead to immune dysfunction and increase the incidence of inflammatory diseases such as metritis and mastitis (1). Calcium, an key mineral and secondary messenger, regulates various cellular physiological functions and signal transduction pathways (2) such as blood coagulation, nerve conduction, membrane permeability, muscle contraction, enzyme activity, hormone release and immune function (3). Studies have shown that hypocalcemia directly impairs the response of immune cells to activating stimuli (4).

Neutrophil (polymorphonuclear, PMNL) are highly-mobile cells that can effectively track, engulf and eliminate microorganisms by producing toxic molecules (5, 6). As a signaling molecule, calcium helps connect multiple PMNL receptors and mediates the interaction of PMNL with the host and pathogens (7). Among cells of the immune system, PMNL play a vital role in the defense against infection in cows afflicted by hypocalcemia (8). Our previous studies have shown that subclinical hypocalcemia can reduce PMNL migration, chemotaxis, adhesion, and phagocytosis in dairy cows during the transition (9). Thus, we hypothesized that calcium imbalance would affect the PMNL transcriptome.

To test the hypothesis, we selected dairy cows with subclinical hypocalcemia according to plasma calcium concentrations. RNA sequencing was used to investigate changes in the transcriptome of circulating PMNL in these cows.

## Materials and methods

Heilongjiang Bayi Agricultural University (Daqing, China) Animal Use and Care Ethics Committee approved the animal research program (No. 2020120701).

### Animals

Holstein dairy cows from a commercial dairy farm in Heilongjiang Province (China) were examined by the attending veterinarian to ensure that there were no other complications: normal calf delivery, absence of placental retention, subclinical mastitis (by California Mastitis Test) or ketosis [blood β -hydroxybutyrate <1.2 mmol/L as measured by hemoketometer (TNN-2, Beijing Yicheng Bioelectronic Technology Co., LTD.)]. Subsequently, blood samples were taken before feeding and after milking. One set of samples (5 mL) was collected into a heparin-sodium tube and plasma isolated after centrifugation at 1,400 × g for 10 min. Plasma calcium concentration was immediately determined using an automatic biochemical analyzer (Pointcare M4, China), which generates results within 10 min. Another set of samples (20 mL) was then collected into a sodium-citrate tube for PMNL isolation. A total of 16 cows were included, among which 8 cows with calcium concentration between 1.38 and 2.00 mmol/L were regarded as low-calcium (LCAL), and 8 cows with Ca^2+^ > 2.10 mmol/L were regarded as controls (CON) (9). All cows had a BCS of approximately 3.25 and 3.50 at calving; CON cows yielded 26.1 ± 3.7 kg and LCAL cows yielded 26.2 ± 4.6 kg, with mean dry matter intake of 20.8 ± 1.4 kg at 2 days postpartum. A portable semi-automatic milking equipment was used to assess milk yield and electronic scales were used to measure output at 06:00 and 15:00 on the sampling days.

### Isolation and culture of PMNL

On day 2 after calving, 5 mL of blood was collected into a tube containing 1 mL of 3.82% sodium citrate (Solarbio, Beijing, China). There were 4 of these tubes collected per cow. After mixing, the sample was transported to the laboratory at 4°C within 2 h. A commercial PMNL isolation kit (Solarbio, Beijing, China) was used for isolation according to the manufacturer's instructions. Briefly, separation solution A and B in the kit and the blood were added into the centrifuge tube according to 4:2:1, and centrifuged at 800 × *g* for 30 min. The sediment was washed and erythrocytes lysed using red cell lysing reagent (Solarbio, Beijing, China) followed by centrifugation at 300 × g for 10 min at 4°C. The PMNL was re-suspended in 1 mL TRIzol reagent (Life Technologies) and stored at −80°C for RNA extraction.

### RNA extraction and sequencing

Total RNA (*n* = 5 in each group) was separated and purified with TRIzol (Invitrogen, Carlsbad, CA, USA) according to the protocols provided by the manufacturer. The NanoDrop ND-1000 (NanoDrop, Wilmington, DE, USA) was used to control the amount and purity of total RNA. The Agilent 2100 (Agilent, USA) was used to test the integrity of RNA, and all samples had an RNA Integrity Number (RIN number) >7.0 (indicating the quality of the samples was appropriate for subsequent mRNA analysis). Total RNA (5 μg) was used for attachment of Dynabeads^TM^ Oligo (dT)25 (Invitrogen, CA, USA) to capture poly(A)mRNA through two rounds of purification. Subsequently, poly(A)mRNA was fragmented into small pieces using divalent cations at high temperature. The cleaved RNA was used for reverse transcription to synthesize cDNA using DNA polymerase I (E. coli) (NEB, MA, USA) and RNase H (NEB, MA, USA). During this process, dUTP Solution (NEB, MA, USA) was incorporated into the two-strands to make the ends of the double-stranded DNA blunt ends. An A-base was then added to the blunt ends of each strand, preparing them for ligation to the indexed adapters. Each adapter contained a T-base overhang for ligating the adapter to the A-tailed fragmented DNA. Agencourt AMPure XP Beads (Beck,an Coulter, CA, USA) were then used to screen and purify the size of its fragments. After treatment of the U-labeled second-stranded DNAs using Uracil-DNA Glycosylase (NEB, MA, USA), a PCR reaction was used to make a library with a fragment size of 300 bp (±50 bp). Lastly, a 150 bp paired-end sequencing on an Illumina Hiseq 4000 (LC Bio, China) was performed following the vendor's recommended protocols. Library construction and sequencing was completed by LC Bio Technology CO., Ltd. Hangzhou, China (https://www.omicstudio.cn/index).

### Gene expression verification

Genes from the RNAseq analysis were randomly selected for verification using fluorescent quantitative PCR in 3 cows not used for RNAseq in each the CON and LCAL groups. Assay information is presented in Table 1. The successfully extracted PMNL was washed in an RNASE-free EP tube. TRIzol was added to each well. After centrifugation with chloroform, RNA was precipitated with isopropyl alcohol (Sigma Aldrich, Beijing, China). After washing with 75% ethanol (prepared with DEPC water), appropriate amount of DEPC water was added and mixed to dissolve RNA. The OD value of the extracted RNA was detected by the nucleic acid protein analyzer (Nabi, MicroDigital, South Korea), and the RNA concentration of the cDNA synthesized by reverse transcription was calculated.

**Table 1:** Primer sequences.

**Gene symbol**	**Primer sequence**	**Amplicon size**	**Gene bank accession number**
β-actin	Forward primer: GCTAACAGTCCGCCTAGAAGCA	403 bp	NM_173979.3
	Reverse primer: GTCATCACCATCGGCAATGAG		
SERPINE1	Forward primer: CAGAAGGTGAAGATTGAGGTG	160 bp	NM_174137.2
	Reverse primer: GGCCCATGAACAGGACAGTTCC		
CLCX12	Forward primer: TCGTGGCAAGGCTGAAGAACAAC	135 bp	NM_001113174.1
	Reverse primer: TCGGGTGGGTCTAGTGGAAAGTC		
MPO	Forward primer: TGGATACCTCGGTGGTGCTGAC	144 bp	XM_005219889.4
	Reverse primer: GCTGCTTGAAGTAGGACAGGAGTTC		
CDKN1A	Forward primer: GACTTAGCAGCAGCAGCAGCAG	144 bp	XM_005223326.4
	Reverse primer: GGACTTCGTAACACCCAGCAGATG		

### RNA isolation and qRT-PCR

RNA was obtained using PrimeScript RT kit of Gdna Eraser (Takara Bio, Dalian, China) to obtain cDNA. PCR reactions (*n* = 3 in each group) were performed in the BioRad iCycler iQTM Real-Time PCR Detection System (Bio-Rad Laboratories Inc., Hercules, CA, USA). Primers used for random validation of bovine transcriptome genes and β-actin are shown in Table 1. The mRNA abundance in each sample was normalized to β-actin and quantified using the 2^−ΔΔ*CT*^ method.

### Sequence and primary analysis

The cutadapt software (https://cutadapt.readthedocs.io/en/stable/, version is cutadapt-1.9) was used to acquire raw sequencing data (*n* = 5 in each group) in fastq format using the parameters: ~cutadapt -a ADAPT1 -A ADAPT2 -o out1.fastq -p out2.fastq in1.fastq in2.fastq -O 5 -m 100. Low-quality sequences and repetitive sequences were removed to generate CleanData in fastq.gz format. CleanData were aligned against the bovine genome (Bos taurus, Ensembl v96) by HISAT2 software (https://daehwankimlab.github.io/hisat2/, version hisat2-2.0.4) to obtain the bam file (the parameter is ~hisat2−1 R1.fastq.gz−2 R2.fastq.gz -S sample_mapped.sam). Then, we used StringTie software (http://ccb.jhu.edu/software/stringtie/, version is stringtie-1.3.4d.Linux_x86_64) to initially assemble genes or transcripts (the parameter was ~stringtie -p 4 -G genome. gtf -o output.gtf -l sample input.bam). Next, all sequences were merged and a comprehensive transcriptome reconstructed using gffcompare software (http://ccb.jhu.edu/software/stringtie/gffcompare.shtml, version: gffcompare-0.9.8. Linux_x86_64). After generating the final transcriptome, StringTie and ballgown (http://www.bioconductor.org/packages/release/bioc/html/ballgown.html) were used to estimate the expression level of all transcripts, calculated as FPKM (FPKM = [total_exon_fragments /mapped_reads(millions) × exon_length(kB)]), (Command line: ~stringtie -e -B -p 4 -G merge.gtf -o samples.gtf samples.bam) with the R package edgeR (https://bioconductor.org/packages/release/bioc/html/edgeR.html) DESeq2 (http://www.bioconductor.org/packages/release/bioc/html/DESeq2.html), and analyze significant differences between samples. Genes with a difference of FC > 2 or FC <0.5 and *p* value < 0.05 were defined as differentially expressed. They were used for GO and KEGG enrichment analyses.

### Ingenuity pathway analysis

The protein interaction network and pathway analysis were generated using STRING (https://cn.string-db.org/) and Cytoscape (https://apps.cytoscape.org/). The genes were grouped into known functional pathways and networks, mainly according to rodent and human data. The significantly different genes (FDR <0.05) resulting from edgeR were uploaded to STRING and Cytoscape. In both cases, default settings were used for simultaneous analysis of up-regulated and down-regulated genes. The analysis focused on pathways involved in development and function of the blood system, as well as networks involved in immune or metabolic diseases.

### Statistical analysis

Data are presented as the means ± standard error of the means (SEM) with *n* representing the number of independent experiments. Data generated from CON and LCAL groups were compared using unpaired Student's t-test. A probability (P) value of <0.05 was considered statistically significant.

## Results and discussion

During the transition period, cows with subclinical hypocalcemia have an increased risk of metabolic and inflammatory diseases including mastitis and metritis. Furthermore, subclinical hypocalcemia induces PMNL dysfunction (10). Changes in PMNL gene expression from pregnancy to lactation have been reported (11); however, the effect of subclinical hypocalcemia on PMNL gene expression *in vivo* is unknown. Here, we aimed to establish a molecular basis for the effect of serum calcium concentration on PMNL dysfunction during the transition period.

**Figure 1:**
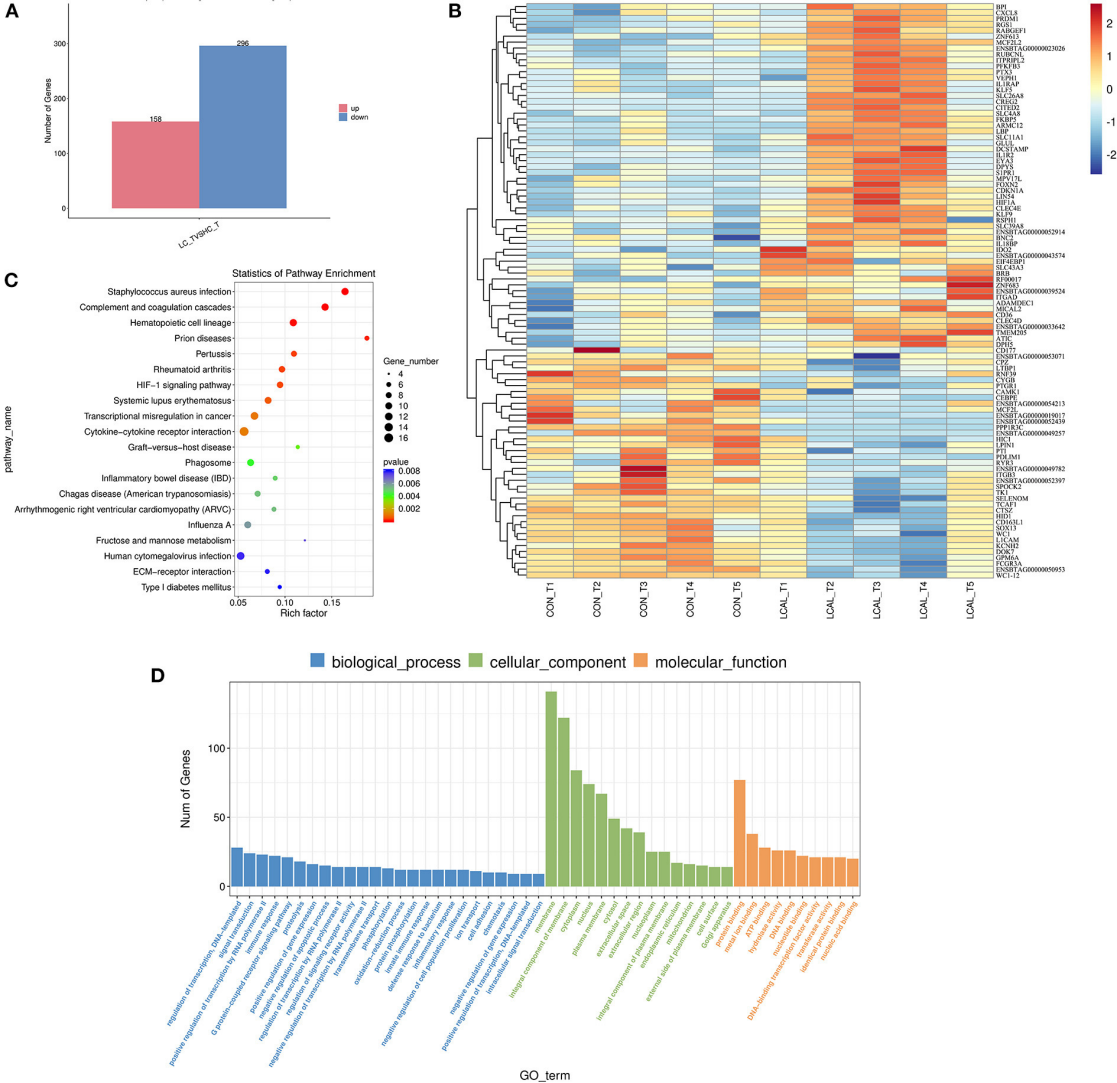
**(A)** Number of differentially expressed genes in PMNL from dairy cows with low plasma calcium [means ± SEM (*n* = 5)]. **(B)** Hierarchical clustering of differentially expressed genes in dairy cows with low plasma calcium. Red represents the up-regulated genes, and blue represents down-regulated genes [means ± SEM (*n* = 5)]. This pathway was generated through the use of omicstudio (https://www.omicstudio.cn/login). **(C)** Ggplot2 was used to display the GO enrichment analysis results in the form of a scatter diagram (bubble diagram). In the horizontal coordinate, Rich factor represents the number of differentially expressed genes located in the GO/the total number of genes located in the GO (Rich factor=S gene number/B gene number) [means ± SEM (*n* = 5)]. This pathway was generated through the use of omicstudio (https://www.omicstudio.cn/login). **(D)** Histogram of GO enrichment analysis of differentially expressed genes in PMNL from dairy cows with low plasma calcium. Data are the analysis of GO Terms enriched in biological processes, cell components and molecular functions. The number of different genes (S gene number) was annotated by GO_Term from largest to smallest, and select GO_Terms include the Top25, Top15, and Top10 differentially expressed [means ± SEM (*n* = 5)].

A total of 454 genes were differentially expressed in LCAL cows, among which 158 genes were up-regulated and 296 genes were down-regulated (Figure 1A). The hierarchical cluster analysis heat map of differential expression is shown in Figure 1B. The histogram of GO enrichment analysis results reflected the distribution of the number of genes with significant differences in GO terms in biological processes, cellular components and molecular function categories in LCAL cows (Figure 1D).

There were 25 genes associated with biological processes involving the regulation of transcription, DNA-template, signal transduction, positive regulation of transcription by RNA polymerase, immune response, and G Protein-coupled receptor signaling. There were 15 cellular component-related genes with significant differences, a response more pronounced than the number of genes associated with biological processes. Among them, the largest numbers of differentially expressed genes include membrane, membrane components, cytoplasm, nucleus, and plasma membrane among others. Regarding molecular function, genes affected are related to protein binding, metal ion binding, ATP binding, hydrolase activity, DNA binding, nucleotide binding, DNA-binding transcription factor activity, transferase activity, identical protein binding, nucleic acid binding.

**Figure 2:**
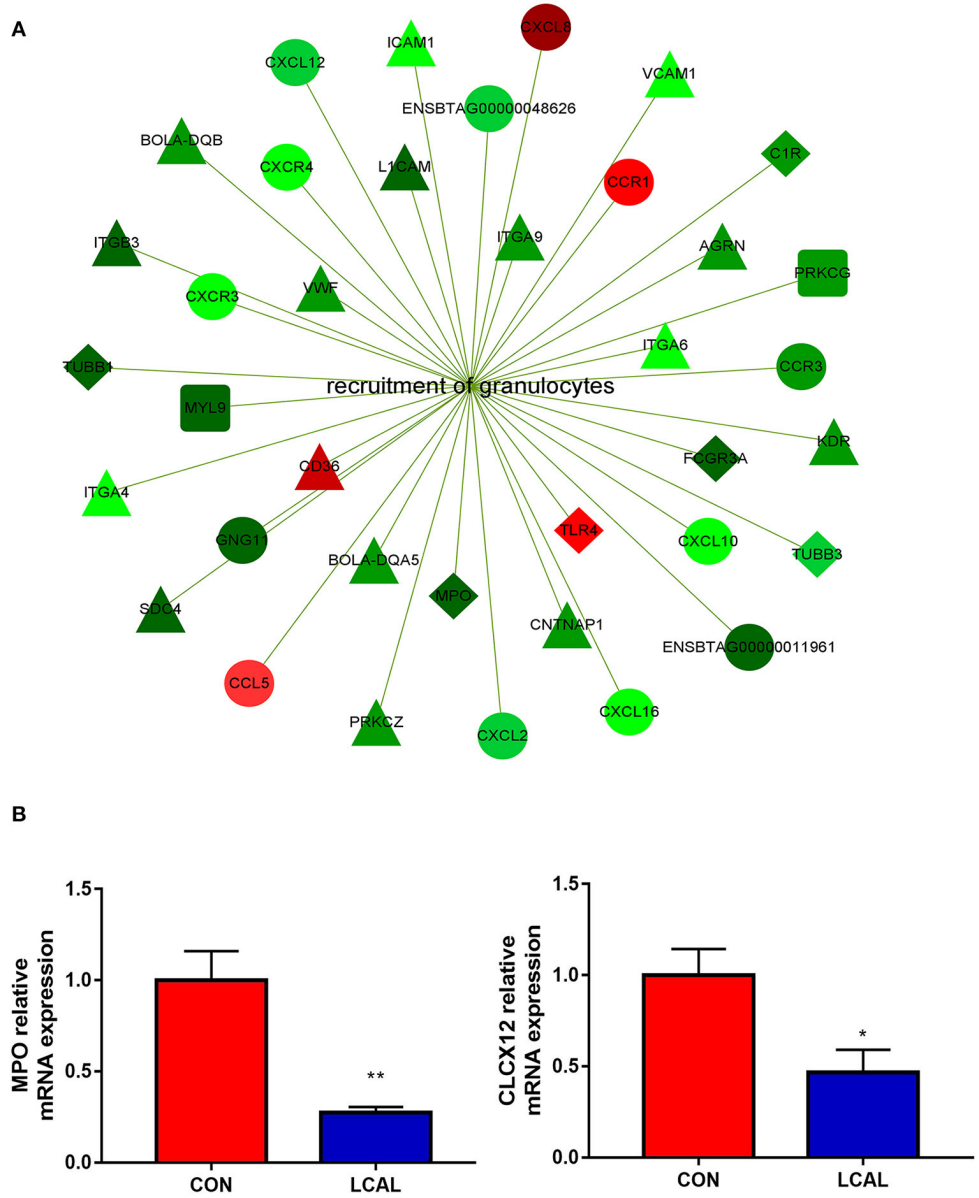
**(A)** Genes related to granulocyte recruitment that were differentially expressed in the PMNL of LCAL group. The color intensity indicates the degree to which the relative expression increased (red) or decreased (green) [mean ± SEM (*n* = 5)]. This pathway was generated through the use of Cytoscape (https://apps.cytoscape.org/). **(B)** Randomly selected genes for verification that are related to granulocyte recruitment [means ± SEM (*n* = 3)].*P < 0.05, **P < 0.01, means there is a significant difference from the comparison table. (one-way analysis of variance).

**Figure 3:**
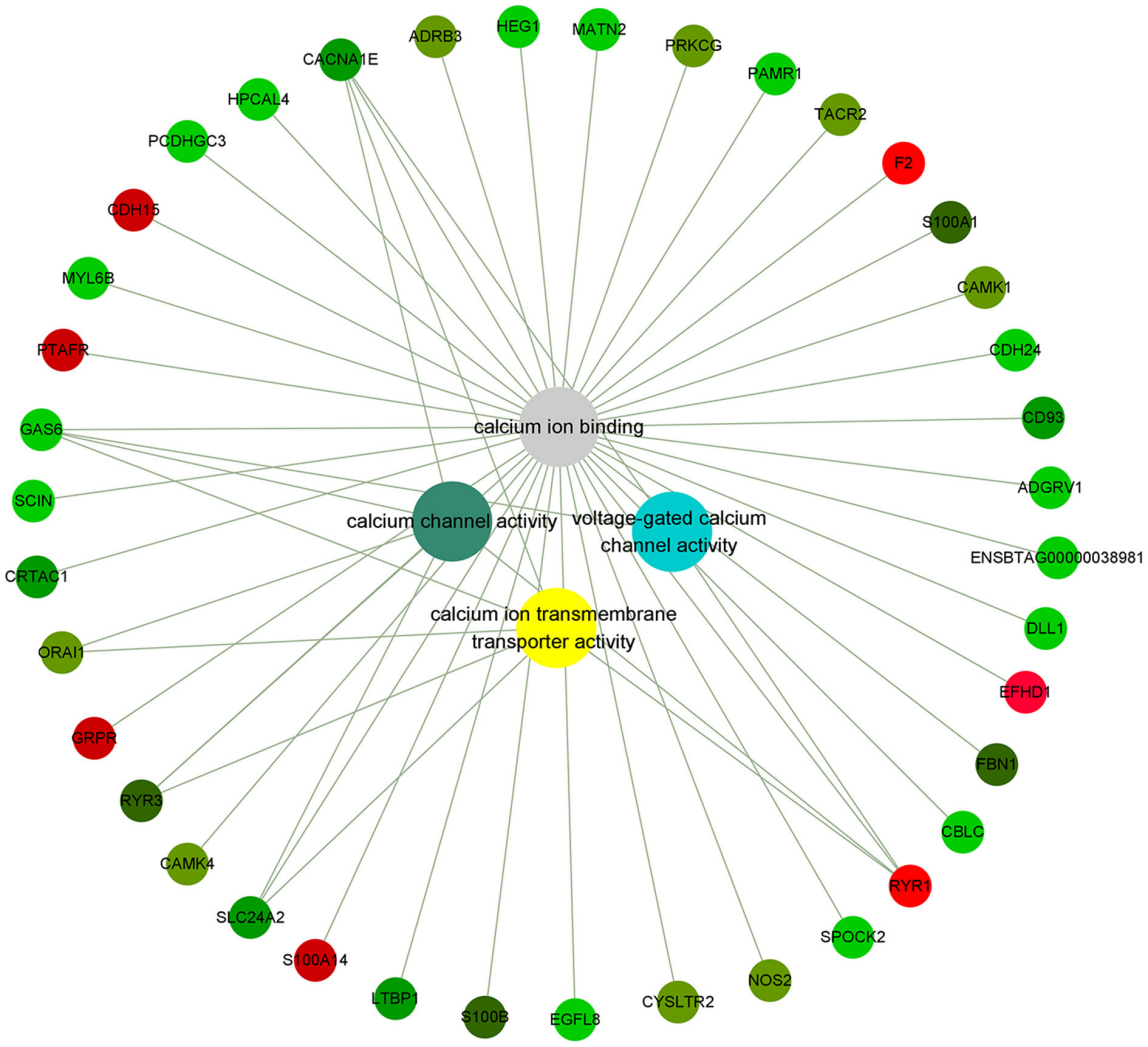
Genes related to calcium ion binding in PMNL that were differentially expressed in LCAL group. The color intensity indicates the degree to which the relative expression increases (red) or decreases (green) [mean ± SEM (*n* = 5)]. This pathway was generated through the use of Cytoscape (https://apps.cytoscape.org/).

**Figure 4:**
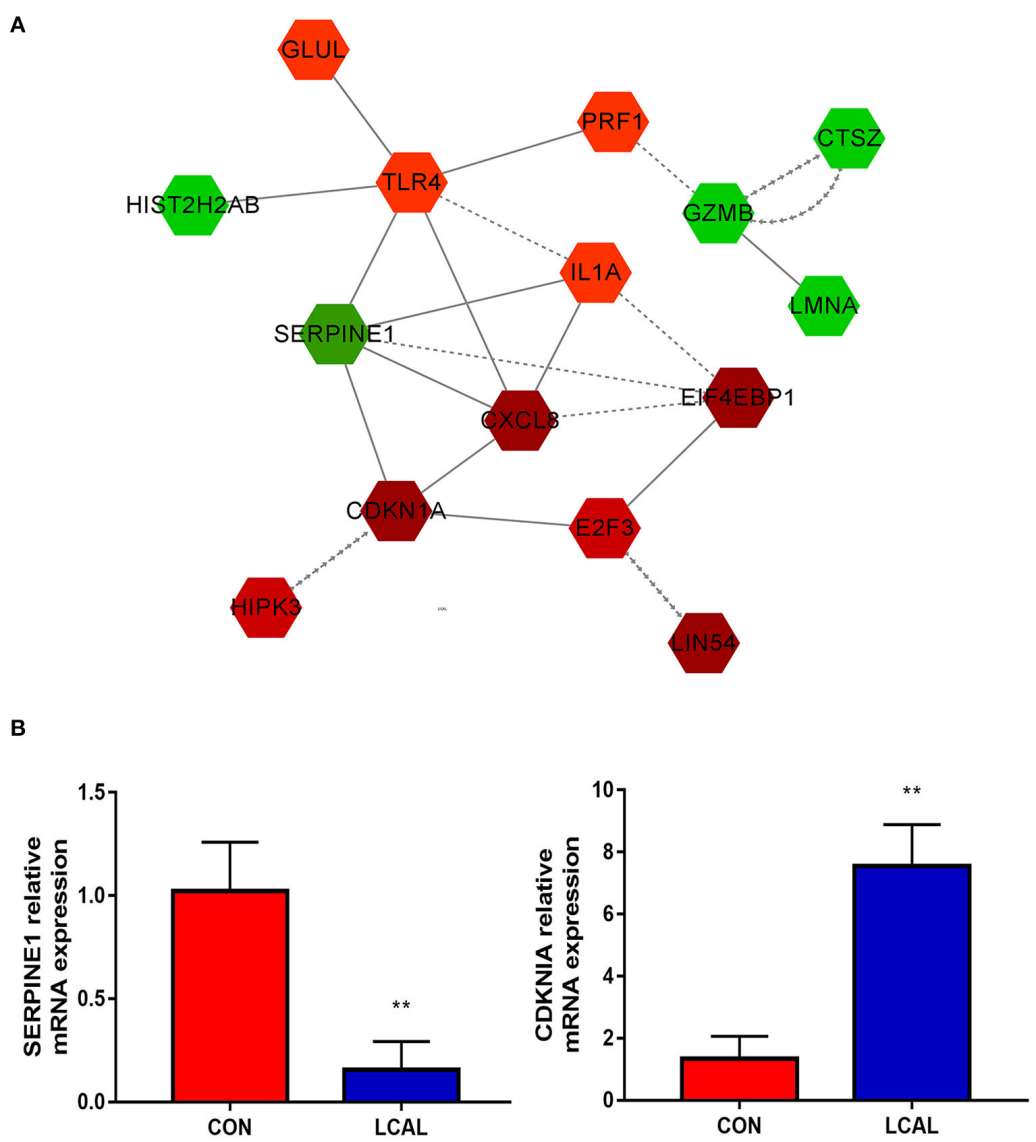
**(A)** Circulating PMNL from LC cows show apoptosis, necroptosis and cell senescence in the process of cell growth and death. The intensity of the color indicates the degree of relative expression increase (red) or decrease (green).and gray indicates no significant changes [mean ± SEM (*n* = 5)]. This pathway was generated through the use of Cytoscape (https://apps.cytoscape.org/). **(B)** Randomly select genes related to granulocyte recruitment for verification [means ± SEM (*n* = 3)]. **P < 0.01 means there is a significant difference from the comparison table. (one-way analysis of variance).

Compared with PMNL in LCAL and CON cows (KEGG database analysis, Figure 1C), Staphylococcus aureus infection, Cytokine-Cytokine receptor interaction, genes related to Phagosome, Fructose and Mannose metabolism were affected. Our analysis showed that these changes in PMNL from LCAL cows were associated with reduced PMNL function (Figures 2–4), which may increase the risk of disease infection in these cows during the transition period (9).

Calcium signaling-related genes were downregulated in PMNL from LCAL cows, indicating that low blood calcium concentrations induced PMNL calcium imbalance. Genes regulating PMNL recruitment and migration, as well as those involved in calcium-related processes, were downregulated in LCAL dairy cows, suggesting that the PMNL inflammatory response also was dysfunctional in these cows (12). PMNL from LCAL dairy cows demonstrated had reduced life span due to an upregulation of genes involved in apoptosis and a decreased expression of genes that regulate cell survival. Complement, which can mediate immune and inflammatory responses, and genes that act as mediators of innate immunity in the coagulation cascade also were down-regulated. Changes were also obvious in the process of infection and inflammation, and may lead to a harmful chronic inflammatory state (13).

### Recruitment of PMNL

Genes involved in the recruitment of PMNL were downregulated in LCAL dairy cows. These included “Chemokine signaling pathway” and “Leukocyte trans-endothelial migration” in the immune system, the “Phagosome” in “Transport and catabolism” and the “Cell adhesion molecule (CAM)” in “signaling molecules and interactions” (Figure 2). In order to enter the site of infection and cause inflammation, host cells and microorganisms release chemotactic agents to guide PMNL to migrate to the area, and PMNL are rapidly recruited to the site of tissue injury to phagocytose and destroy pathogens (14). Thus, the present data agree with previous reports indicating that LCAL can decrease PMNL recruitment and increased risk of mastitis.

Lower expression of CXCL12, CXCL2, CXCL16 and CCR3 in LCAL dairy cows further confirmed a reduced capacity for recruitment and migration of PMNL to the inflammation site (15, 16). LCAL dairy cows had lower expression of PRKCZ, a calcium and diacylglycerol independent serine/threonine-protein kinase, confirming that hypocalcemia reduced PRKCZ kinase activity to decrease transendothelial migration (17, 18), thus, increasing the risk of infectious diseases.

Compared with CON dairy cows, lower expression of MPO, TUBB1, and TUBB3 in LCAL indicated that PMNL phagocytosis and oxidative bursts might have been reduced (19). The expression of adhesion molecules such as L1CAM, member of the immunoglobulin superfamily, was decreased in PMNL of LCAL dairy cows (20). Taken together, the present and previous results suggest that dairy cows with LCAL greatly increase the risk of mastitis partly due to alterations in PMNL gene expression, which causes immune cell dysfunction (21).

### Calcium ion binding pathway

Calcium, an important second messenger, is a critical regulator of multiple physiological processes, including blood coagulation, membrane permeability, and hormone release (22). The impacts of LCAL on dairy cattle welfare and production extend beyond its clinical symptoms. Inadequate concentrations of ionized Calcium in the circulation impaired immune cell function and smooth muscle contraction (4, 23). In LCAL dairy cows, several calcium signaling and calcium-binding genes in PMNL were down-regulated (Figure 3A), which suggest a reduced capacity to respond to calcium as an attempt to attenuate PMNL function (12). These changes included voltage-gated calcium channel activity, calcium ion binding, calcium ion transmembrane transport protein activity, and calcium channel activity related genes. Voltage-gated calcium channels (VGCCs) are transmembrane proteins composed of multiple subunits that translate electrical activities into intracellular calcium elevations and downstream signaling pathways (24). In this study, lower expression of Voltage-dependent L-type calcium channel subunit alpha-1S (CACNA1S; giving rise to L-type calcium currents), CACNA1E (giving rise to R-type calcium currents), and Ryanodine receptor 3 (RYR3; mediating release of Ca^2+^ from the sarcoplasmic reticulum into the cytoplasm in muscle) further confirmed that LCAL cows experienced a decrease in intracellular calcium influx by modulating VGCCs in PMNL. As such, it is likely that chemotaxis and migration capacity were reduced.

Calmodulin plays a key role in the Ca signaling system including the regulation of metabolism and gene expression, normal growth and development of cells, and transport of calcium. As such, one important function related to calcium is that it can promote its absorption. Calcium/calmodulin dependent protein kinase I (CAMK1) and CAMK4 which operate in the calcium-triggered CaMKK-CaMK signaling cascade regulate the activity of several transcription activators, which play pivotal roles in immune response and inflammation. Dependence on Ca^2+^ leads to a significant decrease in the expression of activated small calcium binding protein S100 (S100A1, S100B) (25) genes that influence sarcoplasmic reticulum Ca^2+^ cycling, mitochondrial function, and decreased ROS production all of which could cause immune dysfunction in LCAL dairy cows.

Mitochondria are required for several important processes in PMNL, including chemotaxis, phagocytosis, production of ROS, and regulation of apoptosis. The primary structure of GAS6 contains 4 epidermal growth factor domains, 2 of which contain calcium-binding consensus sequences. GAS6, which has a consensus sequence binding to calcium, was also significantly decreased in LCAL cows. It has been reported that mice with GAS6 gene deletion exhibited abnormal growth of splenic lymphoid organs such as the spleen and lymph nodes and had delayed clearance of apoptotic cells. In addition, they developed a wide-range of autoimmune diseases (26).

### Apoptosis and cell survival

A number of genes differentially expressed between LCAL and CON groups determine changes in cell growth and proliferation, as well as cell death and survival. Circulating PMNL from LCAL dairy cows had decreased cell “apoptosis,” increased “cell senescence” and decreased “necroptosis” during cell growth and death (Figure 4), which is likely to exacerbate inflammatory responses as regulation of PMNL survival/apoptosis is critical for controlling and resolving inflammation. Expression of GZMB, which encodes a potent regulator of cell apoptosis, was decreased in LCAL dairy cows. Expression of cell senescence-related E2F3, CDKN1A and HIPK3 in LCAL dairy cows was significantly increased, suggesting that subclinical hypocalcemia induced cell senescence through increased E2F3, CDKN1A and HIPK3 expression (27, 28). The aging of the organism is accompanied by a series of progressive metabolic changes and the accumulation of senescent cells, as well as the decline of functions and the emergence of various diseases (29). The functional changes of senescent cells come from abnormalities in morphology, quality and their organelles such as mitochondria, endoplasmic reticulum, and cell nucleus (30). The increased senescence of circulating PMNL in subclinical hypocalcemia dairy cows may be another important reason for their susceptibility to inflammatory diseases.

## Conclusions

The results presented here emphasize a link between disequilibrium of calcium homeostasis and peripartal innate immune dysfunction. We have determined that PMNL from dairy cows with subclinical hypocalcemia have an altered transcriptome, including those associated with PMNL recruitment changes, changes in calcium binding, changes in apoptosis and cell survival. These changes indicated reduced immune functions associated with subclinical hypocalcemia, and increased susceptibility to infectious disease during the early transition period for dairy cows.

## Data availability statement

The original contributions presented in the study are included in the article material, further inquiries can be found in the https://doi.org/10.6084/m9.figshare.21078067.

## Ethics statement

The animal study was reviewed and approved by Heilongjiang Bayi Agricultural University (Daqing, China). Written informed consent was obtained from the owners for the participation of their animals in this study.

## Author contributions

BZ, WY, and CX: conceived and designed the experiments. XM, BH, XL, WZ, ML, JNW, YY, and JJW: performed the experiments and analyzed data. BZ and XM: drafted the manuscript. JL and QJ: reviewed and edited. All authors revised final version of the manuscript.

## Funding

The study was supported by grants from the Heilongjiang Province Natural Science Foundation Excellence Youth Project (Grant No. YQ2020C035), National Natural Science Foundation of China (Grant No. U20A2062), and Heilongjiang Bayi Agricultural University Initiating project (Grant No. XYB201809).

## Conflict of interest

The authors declare that the research was conducted in the absence of any commercial or financial relationships that could be construed as a potential conflict of interest.

## Publisher's note

All claims expressed in this article are solely those of the authors and do not necessarily represent those of their affiliated organizations, or those of the publisher, the editors and the reviewers. Any product that may be evaluated in this article, or claim that may be made by its manufacturer, is not guaranteed or endorsed by the publisher.

## References

[1] Neves RC Leno BM Bach KD McArt JAA. Epidemiology of subclinical hypocalcemia in early-lactation Holstein dairy cows: the temporal associations of plasma calcium concentration in the first 4 days in milk with disease and milk production. J Dairy Sci. (2018) 101:9321–31. 10.3168/jds.2018-14587 30077442

[2] Carafoli E. Historical review: mitochondria and calcium: ups and downs of an unusual relationship. Trends Biochem Sci. (2003) 28:175–81. 10.1016/S0968-0004(03)00053-7 12713900

[3] Nestares T López-Aliaga I Alférez MJ Díaz-Castro J Amat A Campos MS. Effect of synergic dietary calcium enrichment and induced ferropenic anemia on antioxidant enzymes activity in rats. Nutrition. (2011) 27:576–81. 10.1016/j.nut.2010.03.009 20591624

[4] Kimura K Reinhardt TA Goff JP. Parturition and hypocalcemia blunts calcium signals in immune cells of dairy cattle. J Dairy Sci. (2006) 89:2588–95. 10.3168/jds.S0022-0302(06)72335-9 16772578

[5] Silvestre-Roig C Hidalgo A Soehnlein O. Neutrophil heterogeneity: implications for homeostasis and pathogenesis. Blood. (2016) 127:2173–81. 10.1182/blood-2016-01-688887 27002116

[6] Oliveira SD Rosowski EE Huttenlocher A. Neutrophil migration in infection and wound repair: going forward in reverse. Nat Rev Immunol. (2006) 16:378–91. 10.1038/nri.2016.49 27231052 PMC5367630

[7] Poplimont H Georgantzoglou A Boulch M Walker HA Sarris M. Neutrophil swarming in damaged tissue is orchestrated by connexins and cooperative calcium alarm signals. Curr Biol. (2020) 30:2761–76. 10.1016/j.cub.2020.05.030 32502410 PMC7372224

[8] LeBlanc SJ. Review: relationships between metabolism and neutrophil function in dairy cows in the peripartum period. Animal. (2020) 14:s44–54. 10.1017/S1751731119003227 32024567

[9] Zhang B Guo H Yang W Li M Zou Y Loor JJ. Effects of ORAI calcium release-activated calcium modulator 1 (ORAI1) on neutrophil activity in dairy cows with subclinical hypocalcemia1. J Anim Sci. (2019) 97:3326–36. 10.1093/jas/skz209 31299068 PMC6667259

[10] Wilkens MR Nelson CD Hernandez LL McArt JAA. Symposium review: transition cow calcium homeostasis-health effects of hypocalcemia and strategies for prevention. J Dairy Sci. (2020) 103:2909–27. 10.3168/jds.2019-17268 31954573

[11] Crookenden MA Erskine CG Kuhn-Sherlock XB Murray A Dukkipati VSR Heiser A. Technical note: evaluation of endogenous control gene expression in bovine neutrophils by reverse-transcription quantitative PCR using microfluidics gene expression arrays. J Dairy Sci. (2017) 100:6763–71. 10.3168/jds.2016-12460 28624280

[12] Zhang B Ma X Loor JJ Jiang Q Guo H Zhang W. Role of ORAI calcium release-activated calcium modulator 1 (ORAI1) on neutrophil extracellular trap formation in dairy cows with subclinical hypocalcemia. J Dairy Sci. (2022) 105:3394–404. 10.3168/jds.2021-21044 35151470

[13] Herrero-Cervera A Soehnlein O Kenne E. Neutrophils in chronic inflammatory diseases. Cell Mol Immunol. (2022) 19:177–91. 10.1038/s41423-021-00832-3 35039631 PMC8803838

[14] Burton JL Erskine RJ. Immunity and mastitis. some new ideas for an old disease. Vet Clin North Am Food Anim Pract. (2003) 19:1–v. 10.1016/S0749-0720(02)00073-7 12682934

[15] Bakogiannis C Sachse M Stamatelopoulos K Stellos K. Platelet-derived chemokines in inflammation and atherosclerosis. Cytokine. (2019) 122:154157. 10.1016/j.cyto.2017.09.013 29198385

[16] Xu X Ye L Zhang Q Shen H Li S Zhang X. Group-2 innate lymphoid cells promote HCC progression through CXCL2-neutrophil-induced immunosuppression. Hepatology. (2021) 74:2526–43. 10.1002/hep.31855 33829508 PMC8597094

[17] Wang Z Yoo YJ De La Torre R Topham C Hanifin J Simpson E. Inverse correlation of TRIM32 and protein kinase C zeta in T Helper Type 2-biased inflammation. J Invest Dermatol. (2021) 141:1297–307 e1293. 10.1016/j.jid.2020.09.021 PMC8058116 33096083

[18] Monick MM Carter AB Flaherty DM Peterson MW Hunninghake GW. Protein kinase C zeta plays a central role in activation of the p42/44 mitogen-activated protein kinase by endotoxin in alveolar macrophages. J Immunol. (2000) 165:4632–9. 10.4049/jimmunol.165.8.4632 11035106

[19] Wang Q Xie Z Zhang W Zhou J Wu Y Zhang M. Myeloperoxidase deletion prevents high-fat diet-induced obesity and insulin resistance. Diabetes. (2014) 63:4172–85. 10.2337/db14-0026 25024373 PMC4238009

[20] Samatov TR Wicklein D Tonevitsky AG. L1CAM: cell adhesion and more. Prog Histochem Cytochem. (2016) 51:25–32. 10.1016/j.proghi.2016.05.001 27267927

[21] Rodríguez EM Arís A Bach A. Associations between subclinical hypocalcemia and postparturient diseases in dairy cows. J Dairy Sci. (2017) 100:7427–34. 10.3168/jds.2016-12210 28690056

[22] Hernández-Castellano LE Hernandez LL Bruckmaier RM. Review: endocrine pathways to regulate calcium homeostasis around parturition and the prevention of hypocalcemia in periparturient dairy cows. Animal. (2020) 14:330–8. 10.1017/S1751731119001605 31337460

[23] Hansen SS Nørgaard P Pedersen C Jørgensen RJ Mellau LS Enemark JD. The effect of subclinical hypocalcaemia induced by Na2EDTA on the feed intake and chewing activity of dairy cows. Vet Res Commun. (2003) 27:193–205. 10.1023/a:1023340506782 12777093

[24] Lazniewska J Weiss N. Glycosylation of voltage-gated calcium channels in health and disease. Biochim Biophys Acta Biomembr. (2019) 1859:662–8. 10.1016/j.bbamem.2017.01.018 28109749

[25] Zimmer DB Weber DJ. The calcium-dependent interaction of S100B with its protein targets. Cardiovasc Psychiatry Neurol. (2010) 2010:728052. 10.1155/2010/728052 20827422 PMC2933916

[26] Lu Q Lemke G. Homeostatic regulation of the immune system by receptor tyrosine kinases of the tyro 3 family. Science. (2001) 293:306–11. 10.1126/science.1061663 11452127

[27] López-Otín C Blasco M Partridge A Serrano L Kroemer MG. The hallmarks of aging. Cell. (2013) 153:1194–217. 10.1016/j.cell.2013.05.039 23746838 PMC3836174

[28] Lopez-Dominguez JA Rodriguez-Lopez S Ahumada-Castro U Desprez PY Konovalenko M Laberge RM. Cdkn1a transcript variant 2 is a marker of aging and cellular senescence. Aging (Albany NY). (2021) 13:13380–92. 10.18632/aging.203110 34035185 PMC8202863

[29] Kwon SM Hong SM Lee YK Min S Yoon G. Metabolic features and regulation in cell senescence. BMB Rep. (2019) 52:5–12. 10.5483/BMBRep.2019.52.1.291 30526768 PMC6386228

[30] Hwang ES Yoon G Kang HT. A comparative analysis of the cell biology of senescence and aging. Cell Mol Life Sci. (2009) 66:2503–24. 10.1007/s00018-009-0034-2 19421842 PMC11115533

